# Immune system challenge improves recognition memory and reverses malaria-induced cognitive impairment in mice

**DOI:** 10.1038/s41598-021-94167-8

**Published:** 2021-07-21

**Authors:** Luciana Pereira de Sousa, Flávia Lima Ribeiro-Gomes, Roberto Farina de Almeida, Tadeu Mello e Souza, Guilherme Loureiro Werneck, Diogo Onofre Souza, Cláudio Tadeu Daniel-Ribeiro

**Affiliations:** 1 Laboratório de Pesquisa em Malária, Instituto Oswaldo Cruz & Centro de Pesquisa, Diagnóstico e Treinamento em Malária (CPD-Mal) of Fundação Oswaldo Cruz (Fiocruz) and of Secretaria de Vigilância em Saúde (SVS), Ministério da Saúde, Fiocruz. Av. Brasil 4365, Manguinhos, Rio de Janeiro, RJ CEP 2104-360 Brazil; 2 Departamento de Bioquímica, Universidade Federal do Rio Grande do Sul (UFRGS), Porto Alegre, Rio Grande do Sul Brazil; 3 Departamento de Epidemiologia of Instituto de Medicina Social, Universidade do Estado do Rio de Janeiro and Instituto de Estudos de Saúde Coletiva da Universidade Federal do Rio de Janeiro, Rio de Janeiro, Brazil; 4 Present Address: Programa de Pós-Graduação em Ciências Biológicas, Instituto de Ciências Exatas e Biológicas, Universidade Federal de Ouro Preto, Minas Gerais, Brazil

**Keywords:** Immunology, Neuroscience, Diseases

## Abstract

The immune system plays a role in the maintenance of healthy neurocognitive function. Different patterns of immune response triggered by distinct stimuli may affect nervous functions through regulatory or deregulatory signals, depending on the properties of the exogenous immunogens. Here, we investigate the effect of immune stimulation on cognitive-behavioural parameters in healthy mice and its impact on cognitive sequelae resulting from non-severe experimental malaria. We show that immune modulation induced by a specific combination of immune stimuli that induce a type 2 immune response can enhance long-term recognition memory in healthy adult mice subjected to novel object recognition task (NORT) and reverse a lack of recognition ability in NORT and anxiety-like behaviour in a light/dark task that result from a single episode of mild *Plasmodium berghei* ANKA malaria. Our findings suggest a potential use of immunogens for boosting and recovering recognition memory that may be impaired by chronic and infectious diseases and by the effects of ageing.

## Introduction

Following antigenic or sensory stimulation, vertebrates undergo changes in the cellular connections of their immune and nervous systems. Therefore, the immune and nervous systems may both be categorized as plastic cognitive systems due to their capacity to recognize real world objects, including microbes, and to their ability to adapt through experience. Interactions between these two systems exist^1–3^, and immunomodulation of the nervous system can occur through either physiological or pathological mechanisms. Previous studies have demonstrated that; (i) exogenous immune stimuli may have positive or negative effects on neuronal plasticity and cognitive performance, depending on the nature and intensity of the immune response elicited^4–6^ and (ii) neurocognitive dysfunction may occur in both human and experimental models of infectious diseases such as malaria^7,8^.

Cerebral malaria (CM), the most severe complication of human malaria caused by *Plasmodium falciparum*^9^, can result in neurocognitive sequelae, including motor deficits, behavioural alterations and severe learning difficulties^7,10–12^. Some of these sequelae are also observed in *Plasmodium berghei* ANKA (*Pb*A) infected C57BL/6 mice, a well-studied model of experimental CM (ECM)^8,13,14^. Cognitive impairment has also been reported in residents of endemic regions presenting with non-severe malaria^15–17^. More recently, we adapted the ECM model in order to assess neurocognitive alterations in mice, and observed neurocognitive impairment following a short-term episode of non-severe malaria treated before brain impairment^18,19^.

Given the known effect of the immune system on neurocognitive function, we hypothesized that immune stimulation may affect cognitive performance. Here, we use *Pb*A infected C57BL/6 mice to evaluate the effects of immune stimuli on behaviour such as memory and anxiety during homeostasis or following a single episode of mild malaria. Our results show a beneficial effect of immune stimulation on cognitive-behavioural parameters in healthy mice and a reversal of cognitive impairment caused by malaria parasite infection.

## Methods

### Mice and parasite

The *Instituto de Ciência e Tecnologia em Biomodelos* of the *Fundação Oswaldo Cruz* (ICTB-*Fiocruz*, Brazil) provided seven-week-old female C57BL/6 mice weighing 20–25 g. Mice were housed in racks with an air filtration system in a room maintained at 25 °C and light/dark cycles of 12 h in cages containing five animals with free access to food and water. All procedures were carried out in accordance with animal welfare practices approved by the Ethical Committee on the Use of Laboratory Animals of *Instituto Oswaldo Cruz* under *CEUA-IOC*: L-010/2015 concession. The study was carried out in compliance with the ARRIVE guidelines. *Plasmodium berghei* ANKA (*Pb*A) infections were carried out using a stable transfected strain of *Pb*A expressing a green fluorescent protein (*Pb*A-GFP) generated as described previously^20^.

### Infection and treatment of experimental groups

Mice were infected intraperitoneally (ip) with 150 μl of thawed cryopreserved *Pb*A-infected red blood cells. Five days after infection, total blood was collected, adjusted to 1 × 10^6^ parasitized erythrocytes in 100 μl of PBS and injected ip to mice in experimental groups. Parasitaemia was monitored by flow cytometry, based on the percentage of GFP^+^ erythrocytes. In this experimental model, the establishment of cerebral malaria (CM) occurs between the fifth and sixth day of infection^19^. Mice were treated on the fourth day of infection (mean parasitaemia 2.5%) with 25 mg/kg of chloroquine (CQ) by gavage for seven days^8^, before any clinical signs of CM. All groups were similarly manipulated. Previous experiments carried out with groups of uninfected mice comparatively treated with CQ or PBS have previously shown that the CQ treatment does not influence the performance of mice in behavioural tasks and/or their anxiety phenotypes^18^.

### Experimental description

**Figure 1:**
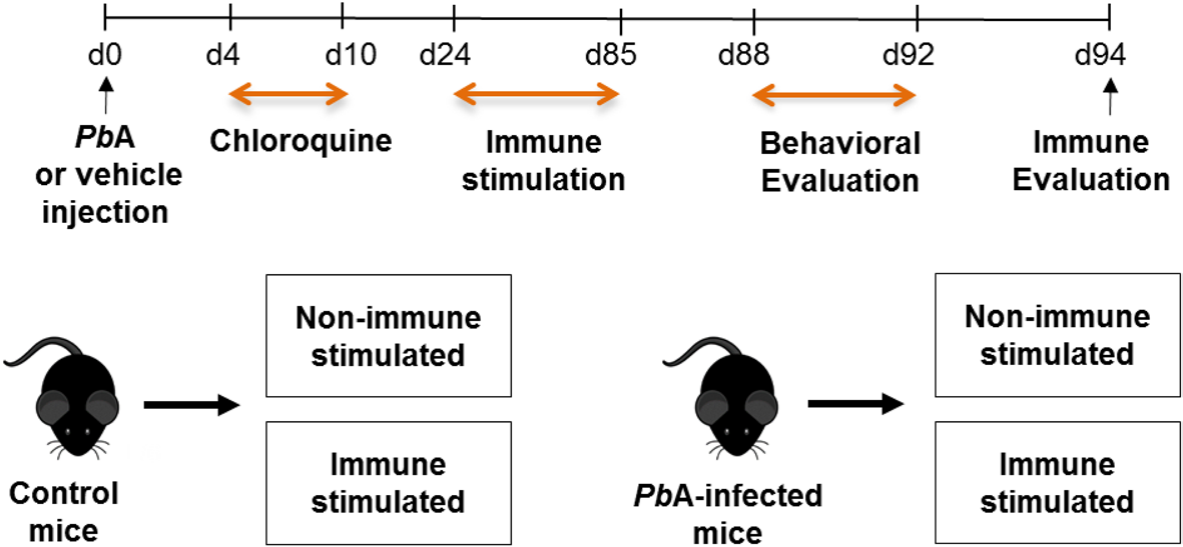
Flowchart of experiments. Groups of mice were infected with *Plasmodium berghei* ANKA (*Pb*A), or mock infected with PBS (Control mice), and treated with chloroquine (25 mg/kg) for 7 days via gavage from the fourth day post-infection. After 14 days, the animals were subdivided into groups of mice immune stimulated with different immunization strategies or non-immune stimulated. Subsequently, mice were evaluated via behavioural tasks for locomotivity, memory and anxiety phenotype. The immune response of randomly chosen mice was evaluated.

Mice were divided into groups of *Pb*A-infected and control animals (non-infected) and both were treated with chloroquine (CQ) for 7 days from the fourth day of infection. Thirteen days after treatment, mice from respective groups were subdivided into non-immune stimulated and immune stimulated groups (Fig. 1). The following vaccines and antigens were used for immune stimulation: Diphtheria and Tetanus toxoids (dT) vaccine for adults, Influenza vaccine, *Plasmodium falciparum* Merozoite Surface Protein 3 (*Pf*MSP-3 recombinant protein), White chicken egg ovalbumin (OVA) and Lipopolysaccharide of *Escherichia coli* (*Ec*LPS). Three different immune stimulation strategies were performed: a combination of all antigens and vaccines described above (henceforth referred to as ‘Pool’); a combination of antigens and vaccines (Influenza vaccine and *Ec*LPS) that trigger, preferentially, a type 1 pattern of immune response (henceforth denominated ‘T1’); and a combination of antigens and vaccines (dT vaccine, *Pf*MSP-3 recombinant protein and OVA) that preferentially trigger a type 2 immune response (henceforth called ‘T2’). Mouse groups were named: Control (non-infected/non-immune stimulated); Pool (non-infected/Pool-immune stimulated); T1 (non-infected/T1-Immune stimulated); T2 (non-infectedimmune stimulated); Inf (infected/non-immune stimulated); Inf-Pool (infected/Pool-immune stimulated); Inf-T1 (infected/T1-immune stimulated) and Inf-T2 (infected/T2-immune stimulated). All control groups were treated as the experimental groups: they were age-matched, mock-immune stimulated, mock-infected, and treated with CQ whenever appropriate. Subsequently, mice behavioural performance was assessed by open field task (OFT), novel object recognition task (NORT) and light–dark task (Fig. S1). About 300 mice were used for these experimental strategies in five consecutive sessions.

### Immune system stimuli

Immune stimulation was initiated 14 days after the end of CQ treatment, and was performed in the course of the following 62 days (Fig. S1). Antigens and/or vaccines were administered by different routes and in different regions of the animal’s body (Table S1). In view of the originality of our approach and the lack of information on immunization procedures influencing mice behaviour, immunogens were chosen according to the pattern of immune response induced and to the accessibility of experimental or human vaccines to our laboratory. The doses administrated were defined based on dose–response protocols available in the literature capable of stimulating the murine immune system without the risk of death^21–25^.

#### Immune stimulation with *Plasmodium falciparum* Merozoite Surface Protein 3 (PfMSP-3 recombinant protein)

Mice were challenged with 10 μg of *Pf*MSP-3/mouse recombinant protein (in collaboration with Clinical Trials of Malaria Vaccines, Vac4All Initiative, Paris, France) adsorbed on 70% adjuvant solution MONTANIDE ISA 50 V2W/O (SEPPIC. Air Liquide—Healthcare), in 100 μl of PBS. Three subcutaneous injections were performed at the tail region with a twenty-day interval between immune stimulations^23^ (Fig. S1, Table S1).

#### Immune stimulation with Tetanus-Diphtheria and influenza vaccines

The vaccines used in this study were: Tetanus-Diphtheria (dT) double bacterial (Biological E Limited—BE, Telangana—India, Lot. 34005815), in collaboration with the Division of Health Surveillance—CAP 3.1 of the *Fundação Oswaldo Cruz* (Rio de Janeiro, Brazil); and Trivalent Influenza granted by the Technological Development and Production Division of *Instituto Butantan* (São Paulo, Brazil, Lot. 160034). Mice received 100 μl (1/5 of the human dose) of dT and Influenza vaccines by subcutaneous (dorsal region) and intramuscular (left quadriceps region) routes, respectively (Table S1). Three inoculations with a twenty-day interval between immune stimulations were performed^21^ (Fig. S1).

#### Immune stimulation with ovalbumin (allergen)

Mice received 50 μg/mice of white chicken egg ovalbumin (SIGMA-ALDERICH, Cod. A5503-50 g) adsorbed onto aluminum hydroxide [Al (OH) 3] in a final volume of 200 μl per animal in three inoculations. The first inoculation was performed at the dorsal region by subcutaneous injection and the following (second and third inoculation) by ip route (Table S1) with 6 days between them^24^ (Fig. S1).

#### Immune stimulation with lipopolysaccharide from *Escherichia coli* (EcLPS)

Mice were challenged with 0.1 mg/kg of *Ec*LPS O111: B4 (SIGMA-ALDERICH, L2630-10MG, Lot 025M4040V12140701) diluted in phosphate-buffered saline (PBS). Two ip inoculations were performed (Table S1) with a range of 9 days between the immune stimulations^25^ (Fig. S1).

### Evaluation of the immune response

Following stimulation of the immune system, mice were randomly selected and sacrificed for individual withdrawal of whole blood, via cardiac puncture, and spleen at day 84 after the end of CQ treatment. Serum samples were preserved at − 70 °C. Total IgG antibody response to *Pf*MSP-3 recombinant protein, Tetanus-Diphtheria toxoids (dT) and Influenza vaccines; the serum cytokine profile; the splenic lymphocyte subpopulations; and the response to Ovalbumin sensitization were evaluated to confirm the effectiveness of immune stimuli.

#### Specific antibody responses

The antibody response against *Pf*MSP-3 recombinant protein and Influenza vaccine were determinate by conventional Enzyme-Linked immunosorbent Assay—ELISA^9,10^, and the antibody response against dT vaccine was determined by Toxin Binding Inhibition—ToBI^26^.

#### Cytokine profile

Cytokines in the serum samples were measured with Cytometric Bead Array (CBA) Mouse Th1/Th2/Th17 (BD Biosciences) according to the manufacturer’s instructions. The data were collected on a FACSCANTO II flow cytometer (BD Bioscience) and analysed by FCAP Array Software 3.0 (BD Bioscience).

#### Splenic lymphocyte subpopulations

Individual spleens were removed and mechanically dissociated using a syringe plunger above 70 μm-pore size Falcon cell strainer (BD Biosciences). Red blood cells were lysed using ACK lysing buffer (Sigma). Single-cell suspensions were counted and incubated with anti-Fcγ III/II (CD16/32) receptor Ab (2.4G2) in PBS containing 3% FCS for 15 min, and immunolabelled for 30 min at 4 °C in the dark with the following fluorochrome-conjugated antibodies: PE-Cy7 anti-mouse CD8 (53–6.7), PerCP-Cy5.5 anti-CD3 (145-2c11), APC-H7 anti-mouse CD4 (GK1.5), APC anti-mouse B220 (RA3-6B2), BB515 anti-mouse CD62L (MEL14), APC anti-mouse CD44 (IM7) and/or PE anti-mouse CD25 (7D4). For Treg cells analyses, cells were fixed and permeabilized, after staining for surface markers, with eBioscience™ Foxp3/Transcription Factor Staining Buffer Set according to the manufacturer instructions and incubated with the antibody Alexa Flour 647 anti-Foxp3 (R16715). All antibodies were from BD Biosciences. Data were collected using FACSDiva software on a FACSCANTO II flow cytometer (BD Biosciences), and analysed using FlowJo software 10.0 (BD Biosciences).

#### Intradermal skin test

In the footpad of the left paw, 3 µg of OVA, diluted in 30 µl of PBS, were injected in each animal. After 30 min, the plantar thickness (mm) was measured using a digital caliper. Oedema formation was expressed as the difference of the pad thickness measured before and after the inoculation of OVA^24^.

### Behavioural analysis

The schedule of the behavioural tasks is shown in Supplementary Fig. S1. Mice were individually submitted to different behavioural paradigms to evaluate their exploratory and locomotor activity, cognitive abilities, and parameters involved in anxiety-like behaviour from day 88 to 92 post-infection (77 to 81 days after the complete parasitological cure of animals obtained with CQ treatment). The beginning of behavioural tests corresponded to 22 days after the last stimulation with *Pf*MSP-3 recombinant protein, Tetanus-Diphtheria, and Influenza vaccines, 7 days after the last injection of ovalbumin, and 2 days after the LPS final inoculation. The same cohort of mice was used in all tasks (Fig. S1). All experiments were carried out with an incandescent light source of 200 lx of intensity in the evening period. Animals were acclimatized in the experimental room for at least two hours before the experimental sessions. Behaviour was captured by a video camera positioned above the task apparatus. Locomotion in the open field and the object recognition task was analyzed by the AnyMaze software 5.1 (Stoelting Co., Wood Dale, IL, USA), while a trained blind-to-treatment researcher evaluated other behavioural parameters by video analysis. In all behavioural tests, mice were individually placed on the apparatus, which was previously cleaned with 70% alcohol and dried.

#### Open field task (OFT)

To address the effect of immune stimuli on locomotion and on long-term habituation, mice were individually submitted to the OFT with a training (OFT1) and a test (OFT2) session 24 h apart, as described elsewhere^18^. In each OFT session, mice were individually allowed to freely explore a grey acrylic square box, dimensions (50 × 50 × 50 cm, length × width × height), for 10 min. In OFT1, locomotor activity (measured by the total distance travelled)  and the time and total distance travelled in the centre zone were evaluated during the entire session. In OFT2, we evaluated the total distance travelled during the session.

#### Novel object recognition task (NORT)

To evaluate long-term memory for object recognition, a NORT was carried out in the OFT apparatus, 24 h after the test session^18^. In the training session, mice were exposed to two identical familiar objects (FO), for which similar exploratory activity was expected^27^. The test session was carried out 24 h later when mice were exposed to a new object (NO) and to one of the FO previously exposed. Memory expression is indicated by the tendency of the animal to spend more time exploring the NO rather that the FO^18,27^. Animals were individually placed in the periphery of the box with the objects in a session for 10 min. Exploration was recorded only when the animals touched the objects, located in opposite and symmetrical corners of the box, with their nose or mouth. The time of exploration of each object was recorded, and its percentage of the time of exploration of both objects was calculated. The object recognition index is calculated as the percentage of time spent on each object (referred to the total time spent on both objects). The difference between the time spent with the NO and the FO is expressed as a delta value obtained with the subtraction of the indexes of each object.

#### Light/dark task

The light/dark task, a conflict avoidance test, was carried out as described by Almeida et al*.*^28^. The apparatus was a rectangular acrylic box (50 × 30 × 30 cm, height × length × width) with two sides coloured white and black, separated by a wall (5 × 5 cm) with an opening at the level of the base of the apparatus joining both sides. A white 100 W lamp, placed 60 cm above the centre of the apparatus, illuminated the white side of the apparatus, while the black side was kept closed without illumination. The mice were individually placed in the light compartment for free exploration of the apparatus for five minutes. The following behavioural parameters were analysed: the time spent in the light compartment and the number of transitions between the compartments (light and dark).

### Statistical analysis

All statistical analyses were performed using a Prism statistical software 7.0 (GraphPad). The data were extracted from the AnyMaze software 5.1. To analyse OFT and light/dark task, we used the absolute data. The time in each object in NORT was transformed into a percentage, from which the delta was extracted based on the subtraction: OF1 − OF2 (training session) and NO − FO (test session). The two-way ANOVA with Bonferroni correct were used to analyse OFT. One-way ANOVA were used to compare multiple groups in the NORT and light–dark task and Student’s t-test for immune response analyses. Data are presented as mean ± standard error. P < 0.05 was considered statistically significant.

## Results

### Type 2 immune stimuli enhance long-term recognition memory in healthy mice

To study the effect of immune stimuli on behavioural paradigms, immunogens were chosen according to the pattern of immune response induced. Three immune stimulation strategies were used: T1 and T2 strategies employed well-known antigens able to induce type 1 and type 2 immune responses (T1—influenza vaccine and LPS; T2—dT vaccine, *Pf*MSP3 and OVA), respectively^21–23,29^. A Pool strategy was created by the combination of T1 and T2 strategies. Briefly, mice were infected with *Pb*A, treated with chloroquine (CQ) and immune stimulated with different strategies according to Fig. 1.

The effects of immune responses on locomotion and long-term spatial habituation were assessed in mice subjected to two different sessions of the open field task (OFT), with training (OF1) and test (OF2) sessions 24 h apart^18^. At the training session, a high rate of locomotor activity is commonly observed. Surprisingly, mice immune stimulated with Pool or T1 strategies showed reduced total OF1 locomotion when compared to non-immune stimulated mice (Fig. S2A).

Commonly, after the training session [first OFT (OF1), exposure], exploratory behaviour decreases as the stress related to novelty disappears, and is usually significantly lower after 10 min of task performance^18,28^. Both non-immune stimulated (Control group) and immune stimulated (Pool, T1 and T2 groups) mice showed decreased locomotion in the test session (Fig. S2A) indicating that immune stimulation did not affect long-term habituation memory.

**Figure 2:**
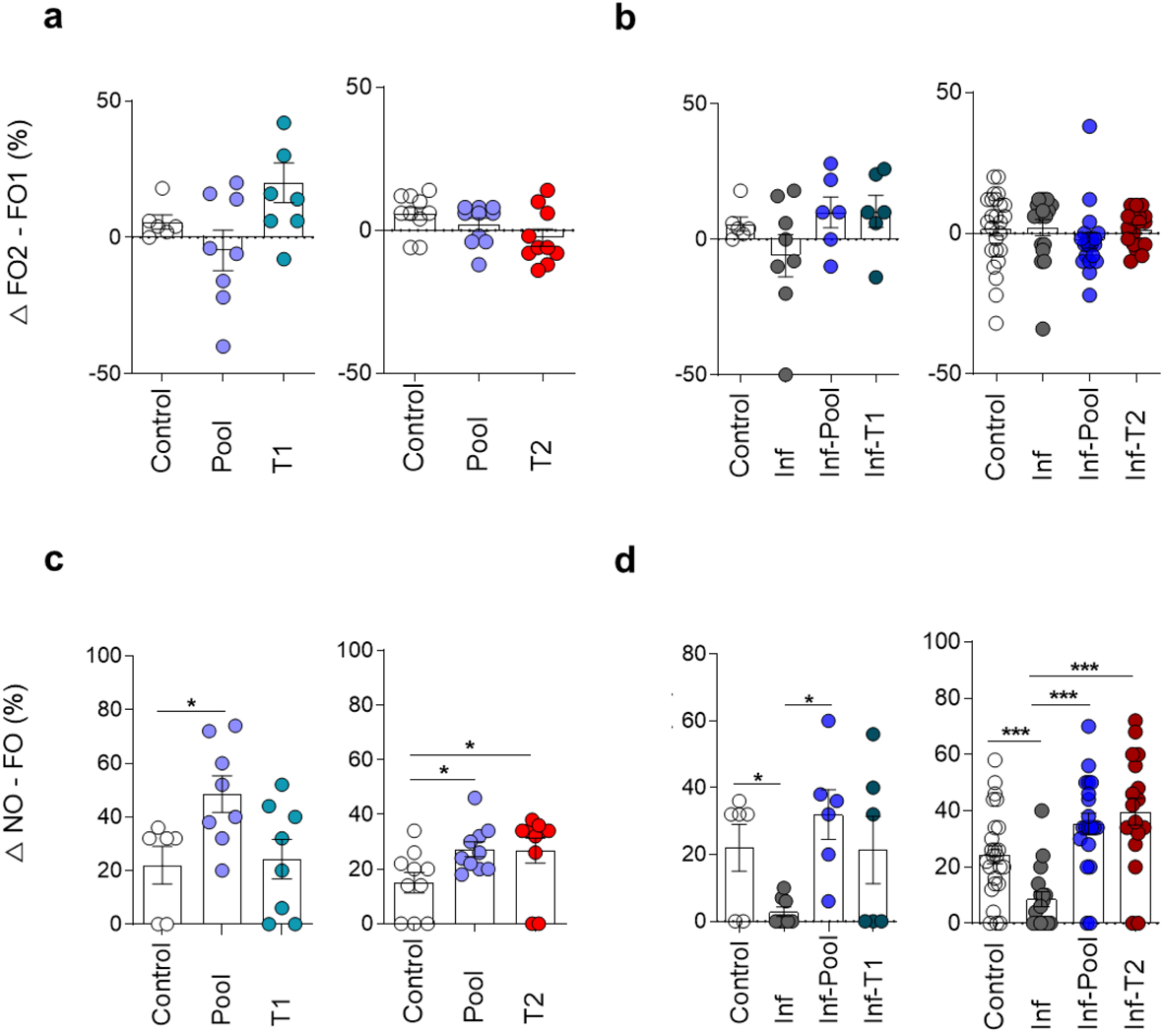
Immune stimulation enhances long-term memory performance in healthy and *Pb*A-infected mice. Healthy or *Pb*A-infected (and treated) mice were immune stimulated, or not, with the Pool, T1 or T2 strategies. Behavioural tasks were performed from day 88 to 92 post-infection (77 to 81 days after CQ treatment). Differences of time, in percentage of total time, spent on each object during the training (**a**,**b**) and test (**c**,**d**) sessions of the new object recognition task (NORT) in healthy (**a**,**c**) and infected (**b**,**d**) mice. *FO* familiar object; *NO* new object. Experimental groups: Control [non-infected/non-immune stimulated mice; n = 6, 10 and 25 (representative of different experiment); Pool (non-infected/Pool-immune stimulated mice, n = 8–10 representative of different experiment); T1 (non-infected/T1-immune stimulated mice, n = 8); T2 (non-infected/T2-immune stimulated mice, n = 10); Inf (infected/non-immune stimulated mice, n = 6–17); Inf-Pool (infected/Pool-immune stimulated mice, n = 6—20); Inf-T1 (infected/T1-immune stimulated mice, n = 6); Inf-T2 (infected/T2-immune stimulated mice, n = 8—18). Data are expressed as mean and s.e.m. ***P < 0.001; **P < 0.01; *P < 0.05; One-Way ANOVA was used [C, F (2,19) = 4.370 and F (2,27) = 3.416; D, F (3,22) = 3.705 and F (3,76) = 12.72]. Data shown represent one of two to five independent experiments (Control, Pool, T1, T2, Inf, Inf-Pool, Inf-T1); and a pool of two independent experiments (Control, Inf, Inf-Pool, Inf-T2).

Next, mice were subjected to the novel object recognition task (NORT) in the same open field arena. The known possible interference of anxiety on memory evaluation is relieved in the NORT sessions by previous exposure in the open field twice on 2 consecutive days. During the training session, a similar exploratory activity of familiar objects (FO1 and FO2) is expected and was observed in all groups of mice (Control, Pool, T1 and T2) (Figs. 2A and S3A), with a mean exploration of 25 s (data not shown). Remarkably, mice immune stimulated with the Pool or T2 strategies presented significantly higher recognition memory performance in relation to the Control group during the test session, reflected by the increased exploitation of the new object in relation to the familiar object, 24 h later. Mice submitted to the T1 strategy did not differ from the Control group (Figs. 2C and S3C). These data indicate that immune stimulation with immunogens that induce type 2 immune responses may enhance long-term recognition memory in healthy mice.

### Immune stimulation of healthy mice did not generate an anxiety-like state

**Figure 3:**
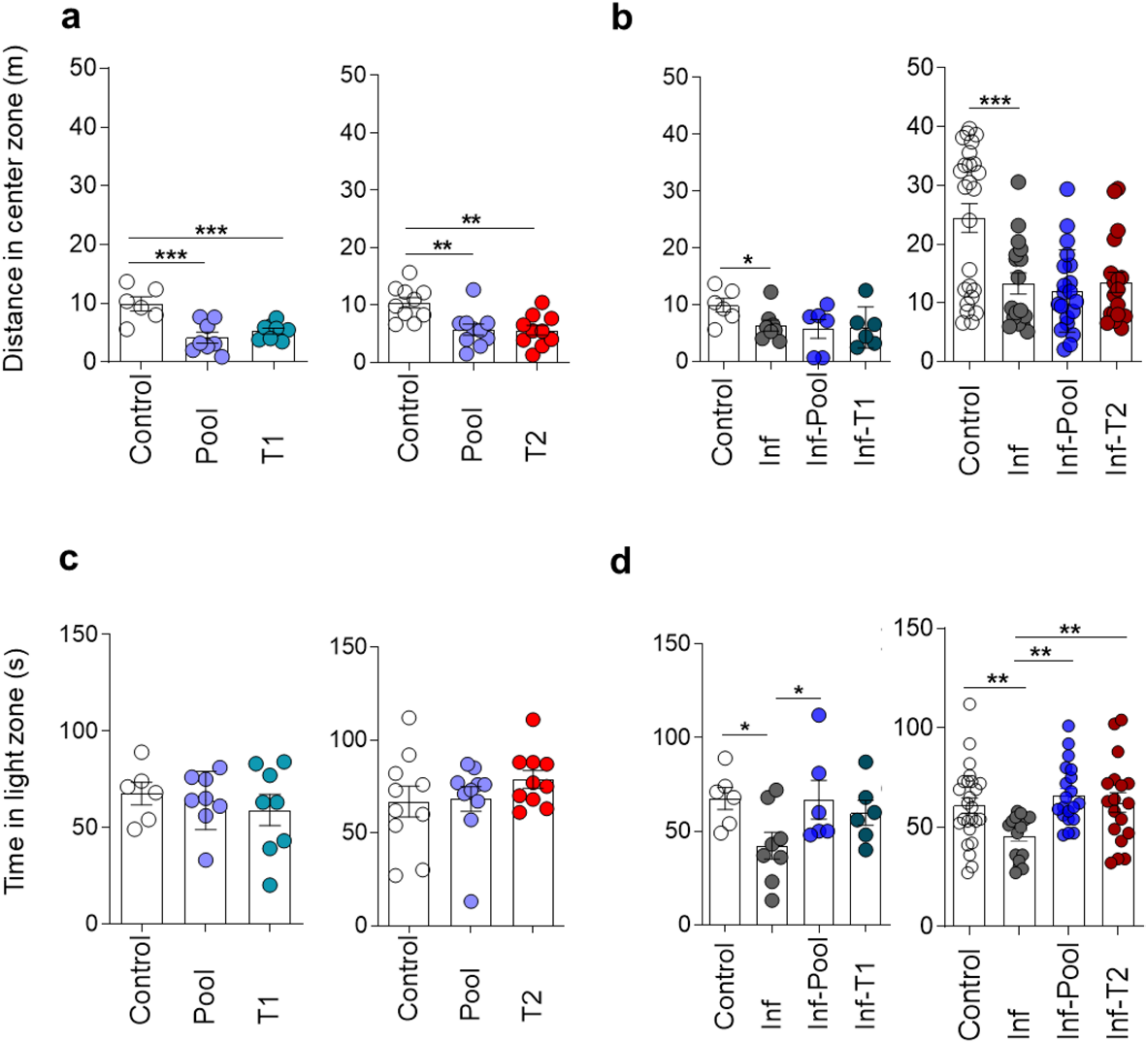
Immune stimulation attenuates the anxiety-like behaviour observed in *Pb*A-infected mice. Healthy or *Pb*A-infected (and treated) mice were immune stimulated, or not, with the Pool, T1 or T2 strategies. Behavioural tasks were performed from day 88 to 92 post-infection (77 to 81 days after CQ treatment). Distance travelled in the centre of the arena during the training session of the open field task (OFT) by healthy (**a**) and infected (**b**) immune stimulated mice. Time in the light zone of the Light/Dark apparatus by healthy (**c**) and infected (**d**) immune stimulated mice. For definition of experimental groups see Legend of Fig. 2. Data are expressed as mean and s.e.m. ***P < 0.001; **P < 0.01; *P < 0.05; One-way ANOVA was used [A, F (2,19) = 11.01 and F (2,27) = 8.901; B, F (3,22) = 3.147 and F (3,76) = 9.175; D, F (3,22) = 3.184 and F (3,76) = 4.727]. Data shown represent one of two to five independent experiments (Control, Pool, T1, T2, Inf, Inf-Pool, Inf-T1); and a pool of two independent experiments (Control, Inf, Inf-Pool, Inf-T2).

In addition to exploratory activity, the OFT also allows the evaluation of phenotypes related to anxiety-like behaviour through analysis of the dwell time or the locomotion rate in the centre of the open field arena during the first exposure to the apparatus. Immune stimulated mice (Pool, T1 and T2 groups) showed no difference in dwell time in the centre of the open field arena (data not shown) but presented significantly reduced locomotion in relation to the non-immune stimulated mice (Control group) (Fig. 3A). It seems, however, that this observation which is consistent with an anxiety-like state, may have been influenced by the total reduced locomotion observed in animals submitted to Pool and T1 strategies (Fig. S2A). In the Light–Dark specific task, however, immune stimulated mice (Pool, T1 and T2 groups) clearly behaved similarly to mice of the Control group, remaining an equal time in the light zone (Fig. 3C), thus implying that immune stimulation did not generate an anxiety-like state.

### Exposure to type 2 immune stimuli may reverse cognitive-behavioural damage induced by non-severe *Plasmodium berghei* ANKA infection

About 98% of the world's malaria cases are due to *Plasmodium falciparum*, 1 to 2% of which progress to CM. Therefore, up to 97% of all malaria cases globally are caused by this lethal parasite, but occur without apparent clinical complications^9^, but may, nevertheless, may impair the cognitive development of children^15–17^.

The experimental model we employed uses *Pb*A-infected C57BL/6 mice treated at day 4 post-infection, before the appearance of any clinical signs of CM^19^. Its main advantage is that it best corresponds to the large majority of human malaria cases in the world; non-severe falciparum malaria^9^. In fact, both parasite-host pairs involve the potential of CM development that can be avoided with timely drug treatment. Using this model, we have been able to observe long-term cognitive-behavioural impairment related to memory and anxiety as late as 92 days post-infection (82 days after the end CQ treatment), when no parasites are present in the blood^18^.

Given the beneficial effect of immune stimulation on long-term memory in healthy mice described above, we evaluated the effect of the same immune stimuli in mice with behavioural alterations caused by non-severe malaria infection. *Pb*A-infected and treated mice (from here on referred to as the “Infected group”), did not display reduced total locomotion in the training session of the OFT when compared to healthy mice (Fig. S2B). However, infected and immune stimulated animals (Inf-Pool and Inf-T2 groups) showed a significant reduction in locomotion in the OF1 when compared to healthy mice (Fig. S2B). Control, infected and infected-immune-stimulated groups (Inf-Pool and Inf-T2, but not Inf-T1) displayed normal behaviour with a significant decrease in locomotion in the test session as compared to the training session of the OFT (Fig. S2B).

As expected, there was no object preference in the NORT training session (Figs. 2B and S3B). Infected mice consistently presented long-term recognition memory sequelae that manifested as similar exploration of the familiar object (FO) and new object (NO) in the NORT. This impairment disappeared following stimuli with Pool or T2 immunization (Figs. 2D and S3D), pointing to a beneficial effect of immune stimulation triggered by type 2 immunogens in reversing the cognitive deficits associated with malaria parasite infection.

The main advantage of the NORT protocol over other rodent memory tasks, for the analysis of cognitive ability is that it relies on the mouse’s natural proclivity for exploring novelty in a well-recognized apparatus. This feature ensures that NORT is much less stressful in comparison with others tasks; and that anxiety does not, therefore, impact NORT performance^27,30^. In this context, the OFT paradigm followed by NORT protocol has been extensively used in preclinical studies to assess different aspects involved in learning and memory, allowing the detection of neuropsychological changes following pharmacological, biological, or genetic manipulations^31^ and the results provided using both of these behavioural paradigms are usually reliable.

### *Plasmodium berghei* ANKA infection in mice induces an anxiety-like behaviour that is reversed by immune stimulation with type 2 immunogens

The distance travelled in the periphery and in the centre of the open field arena are inversely related. Since the latter was decreased in *Pb*A-infected mice (Fig. 3B) and no change in the locomotion during the training session (OF1) occurred among Control and Infected groups (Fig. S2B), the decrease may be interpreted as the expression of an anxiety-like behaviour. This behaviour was confirmed by the observation of a reduction in time spent by infected mice in the light zone of the light–dark task, a more sensitive and widely used test to evaluate anxiety-related parameters in rodents. Anxiety-like behaviour was reversed by Pool and T2, but not by T1 strategies of immune stimulation (Fig. 3D).

### Immune stimulation procedures and non-severe *Plasmodium berghei* ANKA malaria elicit immune responses

Evaluation of specific responses to the immunogens in the Pool, T1 and T2 strategies (tetanus toxoid, influenza, *Pf*MSP3 and OVA) confirmed the effectiveness of the stimuli (Fig. S4A–D) and the known low immunogenicity of diphtheria toxoid in mice^32^.

An effort was also made to characterize systemic inflammatory status at the time of cognitive-behavioural evaluation, through the measurement of TNF-α, INF-γ, IL-6, IL-4, and IL-10 levels in the serum. At the time of immune response evaluation, 84 days after the end of CQ treatment (Fig. 1), non-immune stimulated infected animals did not present increased levels of serum cytokines when compared to the Control group (Fig. S5A–D). However, higher levels of TNFα, IFNγ, IL-6, IL-10 and/or IL-4 were detectable in all groups of immune stimulated mice with T1, T2, or Pool strategies (Fig. S5A–D), ratifying the immune stimulation by the different strategies used. Only IL-10 was consistently increased among healthy and infected mice stimulated with Pool or T2 strategies, although statistical significance was not achieved between Pool and Control groups (Fig. S5E).

We evaluated the splenic immune response of healthy and infected mice exposed to Pool and T2 strategies, since only these approaches were able to immunomodulate cognitive behaviour in mice. Only immune stimulated mice showed increased spleen weight and total number of splenocytes (Fig. S6A–C). Healthy mice immune stimulated with either Pool or T2 strategies presented similar patterns of modulation of different immune components. An increase in the frequency of splenic B cells (Fig. S7B), CD4 and CD8 T cells with central memory phenotype (Fig. S8D,G) and CD4 T cells with regulatory function (Treg cells) (Fig. S7E) were recorded in both Pool and T2 immune stimulated groups when compared to non-immune stimulated animals. A reduction in the frequency of CD8 T cells was also observed in mice immune stimulated with the T2 strategy when compared to the Control group (Fig. S7D).

*Pb*A-infected mice had higher frequencies of B cells, total CD4 and CD8 T cells (Fig. S7A–C), CD4 and CD8 T cells with naïve and central memory phenotypes (Fig. S8A,B,D,E,G) and reduced frequencies of effector/effector memory CD8 T cells (Fig. S8F) when compared to healthy mice (Control group). The frequency of Treg cells, however, was similar between infected and healthy mice (Fig. S7E). Immune stimulation of *Pb*A-infected mice with Pool or T2 strategies induced comparable increases in the frequencies of splenic B cells, Treg cells (Fig. S7A,B,E), effector/effector memory CD4 T cells and central memory CD8 T cells (Fig. S8A,C,G), and reduction in the frequencies of total CD8 T cells when compared to non-immune stimulated infected mice (Fig. S7A,D).

In summary, independent of the health status of the mice, immune stimulation with type 2 immunogens reduces the frequency of CD8 T cells and increases the percentage of Treg cells in the spleen, as well as the serum levels of IL-10.

## Discussion

We demonstrate a clear positive influence of immune responses induced by strategies involving type 2 stimuli on the long-term recognition memory of healthy mice and confirm our previous demonstration of late neurocognitive behavioural dysfunction following a single episode of non-severe malaria^18^. Additionally, our data reveal that immune stimulation with type 2 immunogens subsequent to infection can reverse the malaria induced cognitive impairment.

Stimulation of the immune system can trigger both beneficial or deleterious effects on brain functionality^4–6^. Remarkably, we observed a positive effect of immune stimulation on reversing the cognitive-behavioural impairment associated with non-severe malaria. Mice treated with CQ 4 days after infection by *Pb*A and immune stimulated with T2 and Pool strategies did not display deficits in object recognition that are recorded late after infection without subsequent immune stimulation. We also observed reversal of anxiety-like behaviour in a light–dark task, following immune stimulation of infected mice.

The impairment of recognition (non-spatial) memory, as measured through NORT following a single episode of non-severe malaria, cannot be extrapolated to other memory categories (spatial, emotional, associative) that were not studied here. However, although habituation in the open field task (OFT) is not as robust as the water maze task to evaluate spatial memory, no alteration was recorded in the OFT in the experimental groups evaluated.

Mice presented a clear anxiety-like behaviour in the light–dark task 90 days after a treated episode of non-severe malaria. This result, together with the exploratory behaviour in the OFT centre zone, corroborates those of Guha *et. al.* (2014)^33^, who detected anxious behaviour in another experimental model also considered as non-severe malaria (C57BL/6 mice infected with *Plasmodium chabaudi adami*). It is known that the emotional state of animals may influence exploratory behaviour in tests used to evaluate memory mechanisms. Some acquired concepts weaken the possibility that this may have influenced our results: (a) beyond the investigation of habituation memory in the OFT, it is noteworthy that mice submitted to two OFT sessions before NORT had the anxiety-like phenotype interference mitigated in NORT sessions, as adopted in our protocol and described previously^27,30,31^; (b) the decrease in the total distance travelled in the OFT1 central area by *Pb*A-infected mice is no longer observed in the OFT2 exposition (data not shown), reinforcing that in the second OFT session the anxiety emotional state was abolished; and (c) although locomotion in OFT (centre zone) alone does not directly reflect anxiety behaviour, it may be considered indirect evidence of this emotional condition. In this respect, T1-, but not T2-, treated infected animals showed decreased locomotor activity in the OFT, without presenting memory deficits in the NORT.

It is thought that certain infectious diseases may induce cognitive and behavioural deficits^34–36^. Malaria is a systemic disorder that can affect the central nervous system and the diseases caused by *Plasmodium* species capable of affecting the brain can affect the cerebellum, hippocampus, olfactory bulb and the cerebral cortex, both in humans^37–39^ and experimental models^40–43^. The hippocampus is involved in memory consolidation and anxiety. Additionally, the perirhinal cortex is the centre of familiarity and recency discrimination system^44^. Further studies may clarify whether these or even other brain structures are involved in the present results.

Although no breakdown of the blood–brain barrier is observed on the fourth day of *Pb*A infection, brain histopathologic analysis reveals minimal haemorrhage or oedema restricted to focal areas, with the adherence of low numbers of leukocytes^19^. Expression of immunological markers is also recorded 3 days after infection of C57BL/6 mice with *Pb*A manifested as increased expression of PD-1 (programmed cell death protein) in the cerebellum, CTLA-4 (cytotoxic T lymphocyte associated with protein 4) and LAG-3 (lymphocyte activation gene 3: protein encoded by the LAG3 gene) in the hippocampus, as well as reduced expression of CXCL-4 in the hippocampus^37^. These data indicate that the systemic inflammation already in progress at days 3 and 4 of infection^45,46^ could modulate the brain function leading to cognitive behavioural changes detectable even in the absence of clinical signs of patent cerebral commitment.

T cells are essential for normal neurogenesis and cognition^2,47,48^ and may influence the CNS via the production of cytokines. It has been shown that proinflammatory cytokines impair cerebral function and cognition at high pathological concentrations, as those induced during some infections^2^. An exacerbated peripheral inflammatory response may cause M1 microglial activation and provoke the production of proinflammatory cytokines such as TNF-α and IL1-β that may impair cognitive function^49^. Elevated levels of anti-inflammatory/regulatory cytokines such as IL-4 and IL-10 may have the opposite effect, inducing M2 microglial activation and positively influencing cognition^50,51^.

Microglia activation is recorded even before the overwhelming cerebral inflammation and development of the clinical signs of CM, at day 4 post-*Pb*A infection^19,52^. The serum levels of proinflammatory cytokines also increase around 3–4 days after *Pb*A infection in C75BL/6 mice^45,46^. The observation of early behavioural changes (12 days after malaria treatment), points to a potential reversible (and not preventable) effect of the immune stimuli (unpublished observations). This is in accordance with the concept that sufficiently severe systemic inflammation can damage brain functions in the case of individual vulnerabilities or predispositions^53^ even before the appearance of clinical CM. It is possible, therefore, that the late cognitive deficit observed in our studies results from the early activation of immune cells in the central nervous system (CNS) during malaria infection.

Treg cells are a subset of T cells with immunomodulatory function, important for immune and neuronal homeostasis under physiological conditions, and for the control of pathological immune responses^54–56^ They perform their function mainly via secretion of IL-10 and TGFβ, anti-inflammatory/regulatory cytokines^54–56^ as documented in rodent models^45,46^. The neuroprotective activity of Treg cells, through a mechanism that may involve IL-10 secretion, has been described in murine models of Parkinson's disease, HIV-1-associated neurodegeneration, amyotrophic lateral sclerosis and stroke^57–60^. In the present study, healthy and infected mice stimulated with type 2 immunogens significantly increased the number of splenic Treg cells and IL-10 levels in the serum. As Treg cells and IL-10 can restrict neuroinflammation^45,61^, it is reasonable to assume that these immunogens may enhance cognitive function by promoting balanced cross-talk between the immune system and the CNS mediated through Treg cells and IL-10. The mechanism by which immune stimulation with type-2 immunogens benefits cognition is presently under investigation.

## Conclusion

Our data demonstrate the positive influence of immune responses induced by type 2-inducing stimuli on the long-term recognition memory of healthy mice, confirm our previous demonstration of late neurocognitive behavioural dysfunction following a single episode of non-severe malaria^18^, and demonstrate the recovery of this deficit upon immune stimulation with type 2 immunogens following infection. Taken together, these results offer a new paradigm for the design of memory enhancement strategies and suggest that vaccination procedures may provide benefits additional to the prevention of infection, offering a potential approach for boosting and recovering recognition memory that may have been impaired by chronic and infectious diseases, including malaria, and by the effects of ageing.

## Supplementary Information

Supplementary Information.
