# Supplementary material for: Immune system challenge improves recognition memory and reverses malaria-induced cognitive impairment in mice
Source: Sci Rep. 2021 Jul 21;11:14857. doi: 10.1038/s41598-021-94167-8 (PMC8295320; doi:10.1038/s41598-021-94167-8)
Supplement: Supplementary file 1 — Supplementary Information. [file 41598_2021_94167_MOESM1_ESM.pdf]

Scientific Reports - Supplementary Materials

IMMUNE SYSTEM CHALLENGE IMPROVES RECOGNITION MEMORY AND REVERSES MALARIA-INDUCED COGNITIVE IMPAIRMENT IN MICE

Luciana Pereira de Sousa[1#], Flávia Lima Ribeiro-Gomes[1#], Roberto Farina de Almeida[2+], Tadeu Mello e Souza[2], Guilherme Loureiro Werneck[3], Diogo Onofre Gomes de Souza[2] & Cláudio Tadeu Daniel-Ribeiro[1]*

* Corresponding author at Laboratório de Pesquisa em Malária, Instituto Oswaldo Cruz, Fiocruz. Av. Brasil 4365, Manguinhos, Rio de janeiro. CEP 2104-360, RJ Brazil. E-mail: malaria@fiocruz.br

SUPPLEMENTARY FIGURES WITH LEGENDS AND SUPPLEMENTARY TABLE

Figure S1

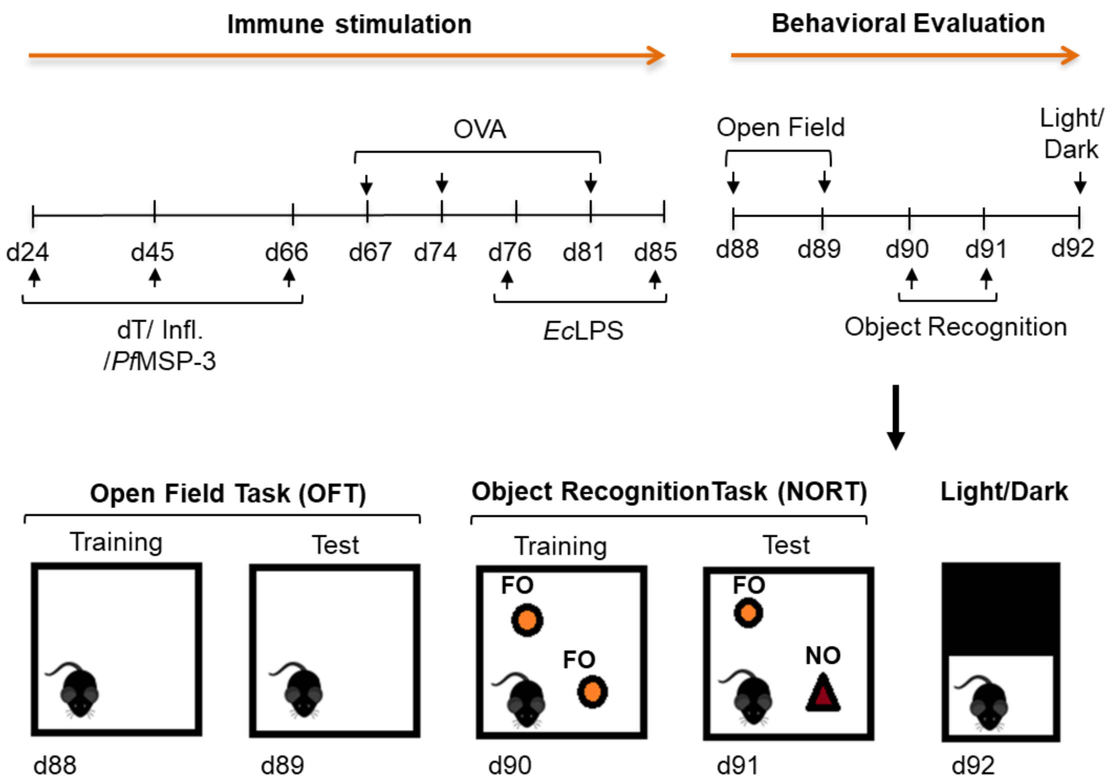

Supplementary Figure 1. Flow chart of immune stimuli and behavioural assessment. Mice (infected or not with *Pb*A and treated with CQ) were immune stimulated or non-immune stimulated, according to the composition of the immunization (Pool, T1 and T2) strategies used. Three doses of the dT and Influenza vaccines and the *Pf*MSP-3 recombinant protein were inoculated conjointly, in different pathways, with a twenty-day interval between inoculations. Three doses of OVA, with a six-day interval between each one, were inoculated one day after the third dose of dT and Influenza vaccines and *Pf*MSP-3 protein. The first injection of *Ec*LPS was done two days after the second dose of OVA, being the second of two injections administered nine days after the first one. Assessment of performance on behavioural tasks started 88 to 92 days post infection (77 to 81 days after the complete parasitological cure of animals obtained with CQ treatment). The beginning of behavioural tests corresponded to 22 days after the last stimulation with the vaccines (Tetanus-Diphtheria and Influenza) and the *Pf*MSP-3 recombinant protein; 7 days after the latter injection of Ovalbumin; and 3 days after the LPS final inoculation. The open field was performed to measure locomotivity, spatial habituation memory and anxiety phenotype, in two sessions [training (OF1) and test (OF2)]. Thereafter, the new object recognition task (NORT) was performed to measure long-term recognition memory, also in two sessions at consecutive days (training with two identical familiar objects and testing with one familiar object and one new object). Finally, the anxious behaviour phenotype was specifically evaluated, by the light-dark test, in a unique session.

Figure S2

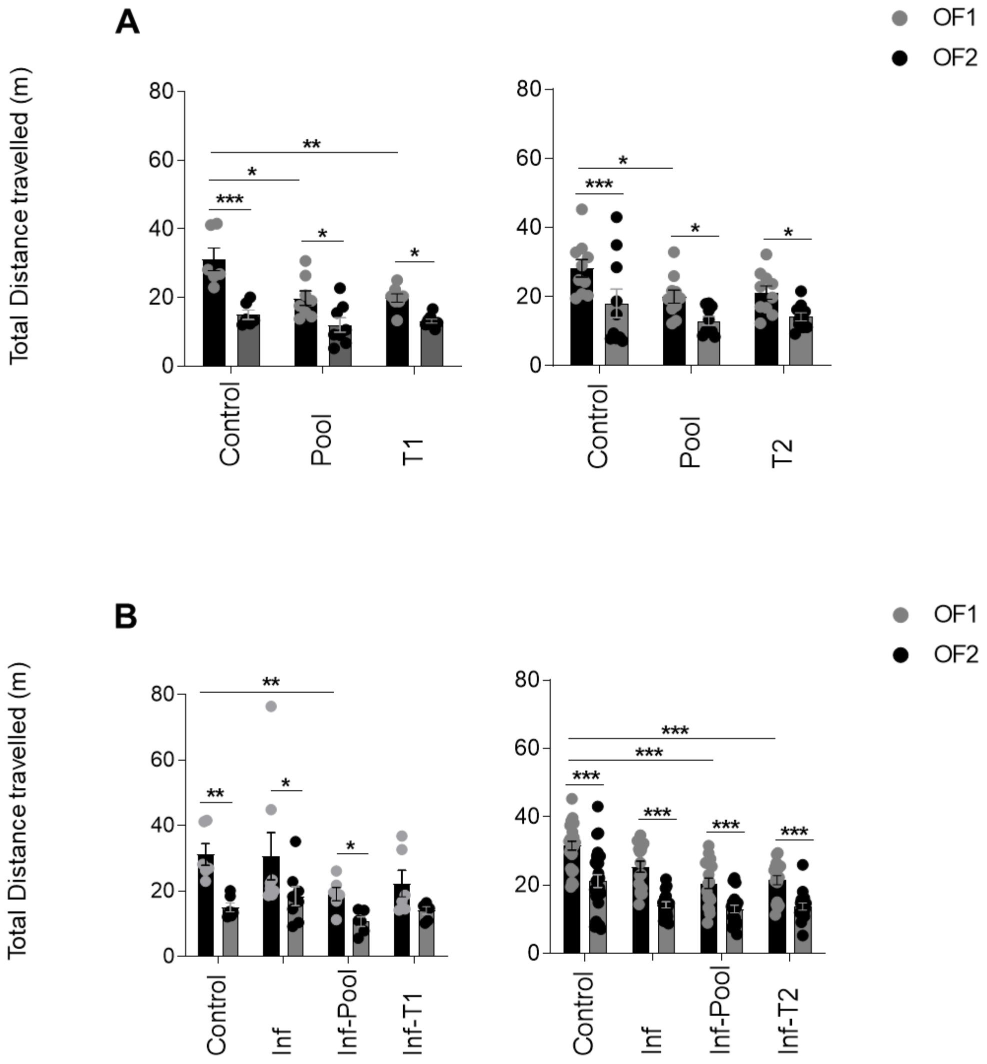

Supplementary Figure 2. Immune stimulation and *Pb*A infection do not influence habituation memory in mice. Healthy or *Pb*A-infected (and treated) mice were immune stimulated, or not, with the Pool, T1 or T2 strategies. Behavioural tasks were performed from day 88 to 92 post-infection (77 to 81 days after CQ treatment). Total distance travelled in the Open Field Task (OFT) during the training (OF1) and test session (OF2) in healthy and infected mice (**A, B**) were evaluated. OFT: healthy mice groups (Control, n = 6; Pool, n = 8; T1, n = 8 and Control, n = 10; Pool, n = 10; T2, n =10) and infected mice groups (Control, n = 6; Inf, n = 8; Inf-Pool, n = 6; Inf-T1, n = 6 and Control, n = 25; Inf, n = 17; Inf-Pool, n = 20; Inf-T2, n = 18). Data shown represent one of two to five independent experiments (Control, Pool, T1, T2, Inf, Inf-Pool, Inf-T1); and a pool of two independent experiments (Control, Inf, Inf-Pool, Inf-T2). Data are expressed as mean and s.e.m. ***$P < 0.001$; **$P < 0.01$; *$P < 0.05$; Two-way ANOVA was used.

Figure S3

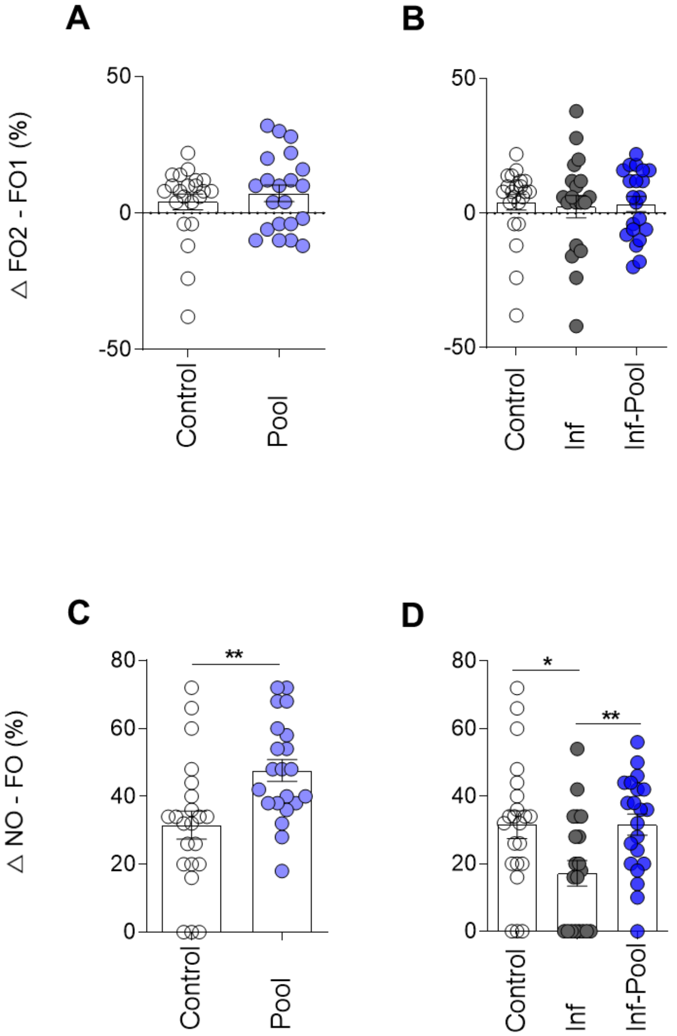

Supplementary Figure 3. Immune stimulation enhances long-term memory performance in healthy and *Pb*A-infected mice. Healthy or *Pb*A-infected (and treated) mice were immune stimulated, or not, with the Pool strategy. Behavioural tasks were performed from day 88 to 92 post-infection (77 to 81 days after CQ treatment). The exploration of the two familiar objects (FO1 and FO2), during the training session of NORT (**A**, **B**), and of the FO and the novel object (NO), during the test session (**C**, **D**), were explored and are expressed as differences in percentage of the exploration time. All groups of mice explored similarly the FO1 and FO2 during the training session (**A**, **B**). Immune stimulation of healthy mice with Pool strategy (Pool group) enhanced the exploratory time spent on the NO in relation to the FO, as compared to the Control group (**c**). Pool-immune stimulation of *Pb*A-infected mice (Inf-Pool group) reversed the memory deficit of *Pb*A-infected mice (Inf group) (**D**). NORT: healthy mice group (Control, n = 22; Pool, n = 21) and infected mice group (Control, n = 22; Inf, n = 20; Inf-Pool, n = 21). Data shown represent a pool of two independent experiments. Data are expressed as mean and s.e.m. ***$P < 0.001$; **$P < 0.01$; *$P < 0.05$; Unpaired t-test was used.

Figure S4

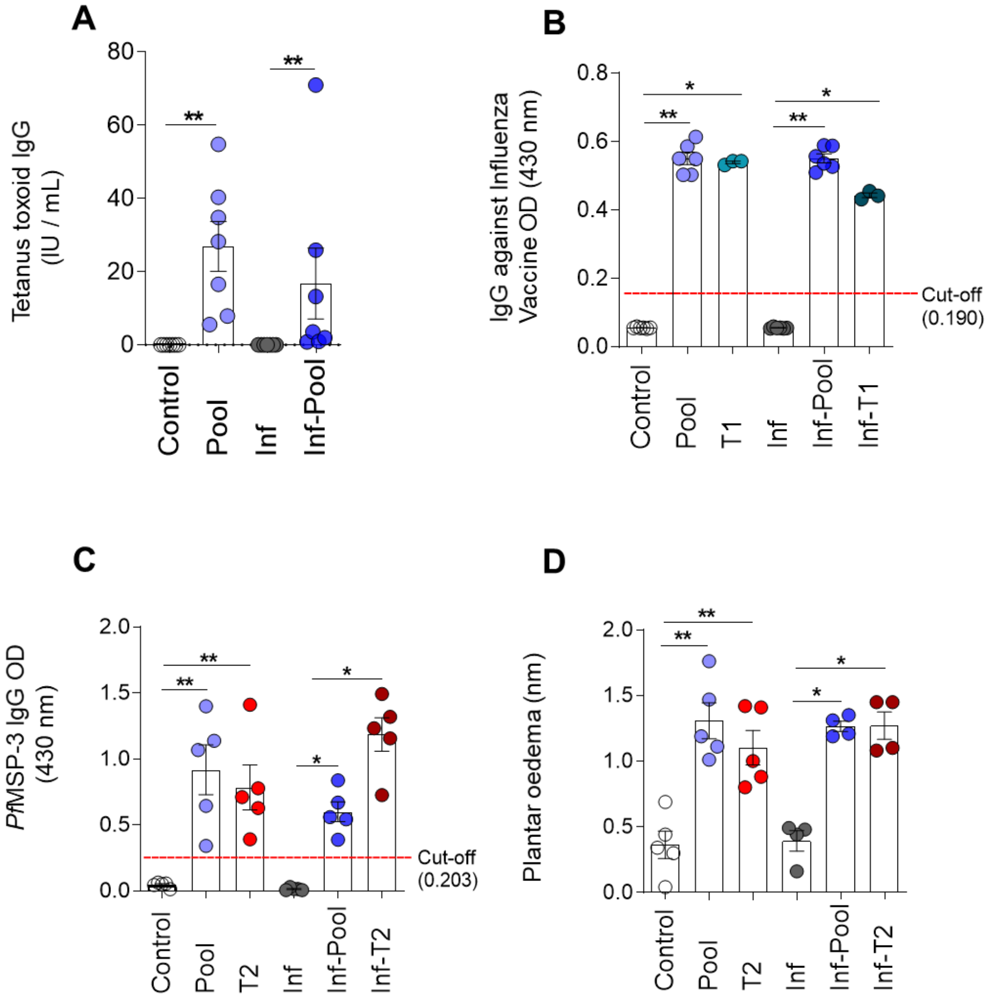

Supplementary Figure 4. Immune stimulation with dT and influenza vaccines, *Pf*MSP-3 and OVA proteins triggers specific immune responses. Healthy or *Pb*A-infected (and treated) mice were immune stimulated, or not, with the strategies: Pool, T1 or T2. After behavioural evaluation, mice were randomly chosen for the analysis of the effectiveness of immune stimulation. Serum levels of (**A**) dT-specific IgG (n = 7), (**B**) Influenza-specific IgG (n = 3 - 6), and (**C**) *Pf*MSP-3-specific IgG (n = 5) were measured. Reaction to OVA was elicited by intradermal injection of the antigen in the footpad of the OVA-sensitized mice (**D**). Oedema was determined by measuring the thickness of the paw before and after inoculation (n = 4 - 5). Experimental groups: Control (non-infected / non-immune stimulated); Pool (non-infected / Pool-immune stimulated); T1 (non-infected / T1-immune stimulated); T2 (non-infected / T2-immune stimulated); Inf (infected / non-immune stimulated); Inf-Pool (infected / Pool-immune stimulated); Inf-T1 (infected / T1-immune stimulated); Inf-T2 (infected / T2-immune stimulated). Data are expressed as mean and s.e.m. ***P* < 0.01; **P* < 0.05; Unpaired t-test with Mann-Whitney test was used.

Figure S5

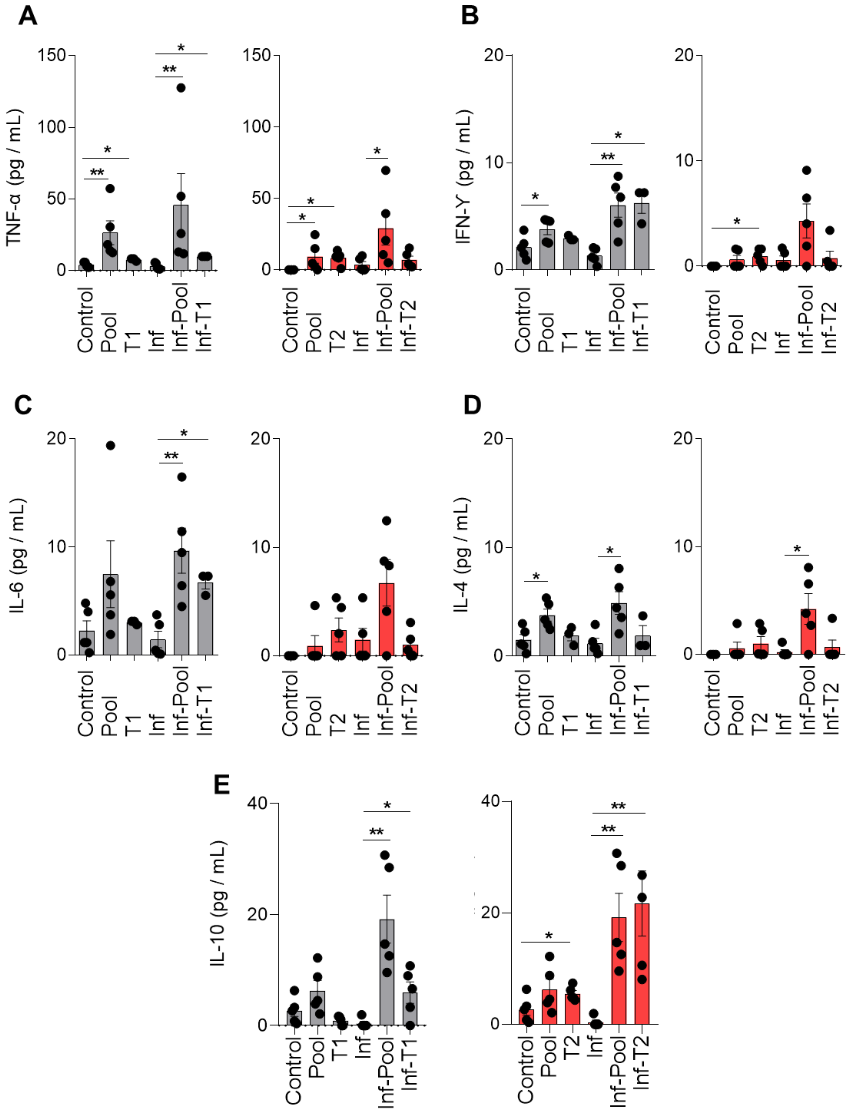

Supplementary Figure 5. Immune stimulation with the different strategies elicits cellular responses measured by increased serum cytokine levels. Healthy or infected (and treated) mice were immune stimulated, or not, with the strategies: Pool, T1 or T2. Serum samples were collected after the behavioural evaluation (84 days after the end of CQ treatment), and levels of the cytokines (**A**) TNFα, (**B**) IFNγ, (**C**) IL-6, (**D**) IL-4 and (**E**) IL-10 were quantified by flow cytometry using cytometric bead array. Experimental groups: Control (non-infected / non-immune stimulated, n = 3 - 5); Pool (non-infected / Pool-immune stimulated, n = 5); T1 (non-infected / T1-immune stimulated, n = 3); T2 (non-infected / T2-immune stimulated, n = 5); Inf (infected / non-immune stimulated, n = 5); Inf-Pool (infected / Pool-immune stimulated, n = 5); Inf-T1 (infected / T1-immune stimulated, n = 3); Inf-T2 (infected / T2-immune stimulated, n = 5). Data are representative of three (Control, Pool, Inf and Inf-Pool groups) and one (T1, T2, Inf-T1 and Inf-T2 groups) independent experiments. Data are expressed as mean and s.e.m. $**P < 0.01$; $*P < 0.05$; Unpaired t-test with Mann-Whitney test was used.

Figure S6

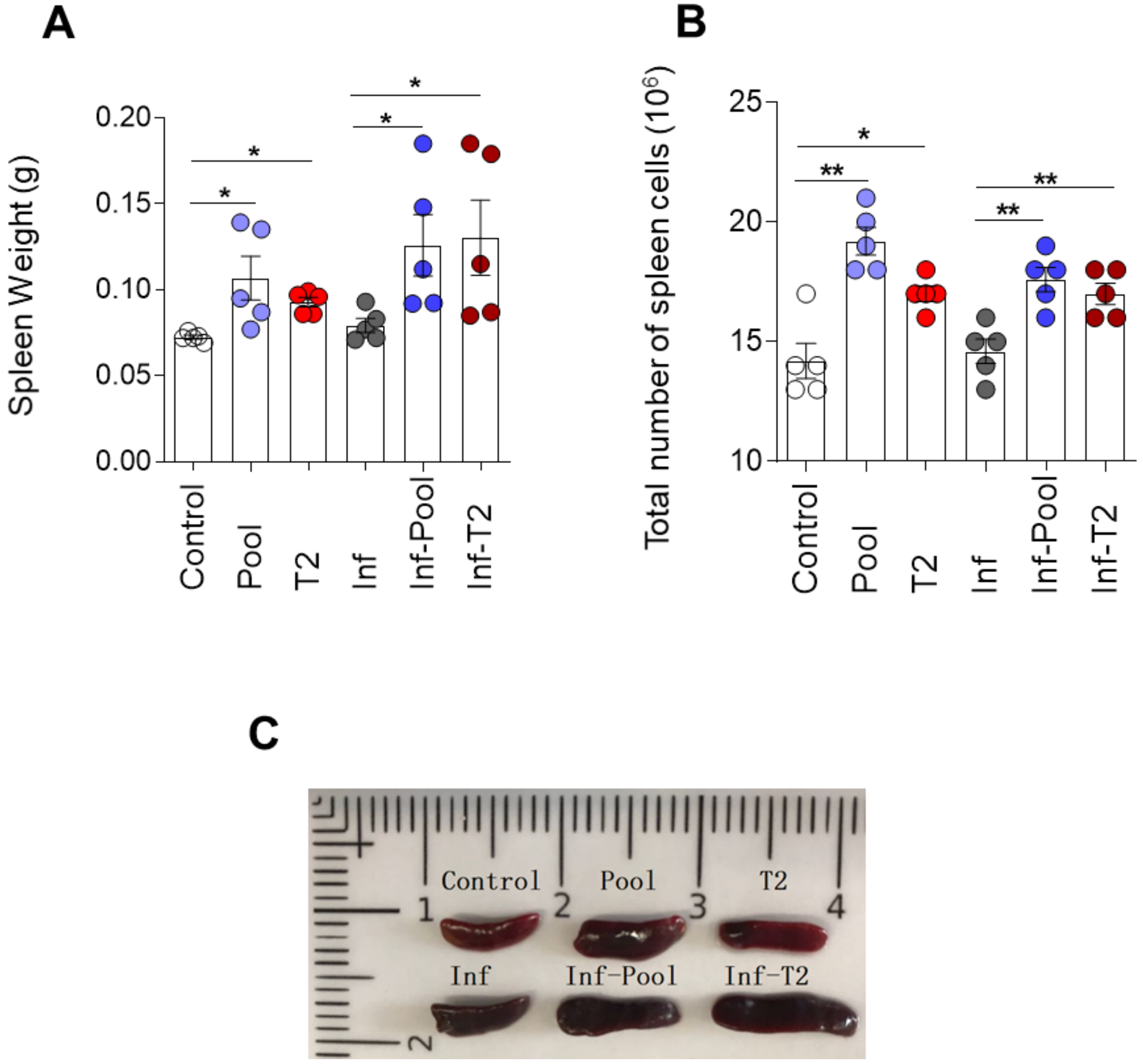

Supplementary Figure 6. Splenic enlargement is observed after immune stimulation. Healthy or infected (and treated) mice were immune stimulated, or not, with the Pool or T2 strategy. Spleen weight (**A**) and total number of splenocytes (**B**) were evaluated at the end of the cognitive behavioural tasks (n = 5) (84 days after the end of CQ treatment). Representative photograph of Control, Pool, T2, Inf, Inf-Pool and Inf-T2 groups (**C**). Groups of infected mice showed a dark colour attributed to hemozoin, even more than two and a half months after infection. Experimental groups: Control (non-infected / non-immune stimulated); Pool (non-infected / Pool-immune stimulated); T2 (non-infected / T2-immune stimulated); Inf (infected / non-immune stimulated); Inf-Pool (infected / Pool-immune stimulated); Inf-T2 (infected / T2-immune stimulated). Data are mean and s.e.m. $^{**}P < 0.01$; $^{*}P < 0.05$; Unpaired t-test with Mann-Whitney test was used.

Figure S7

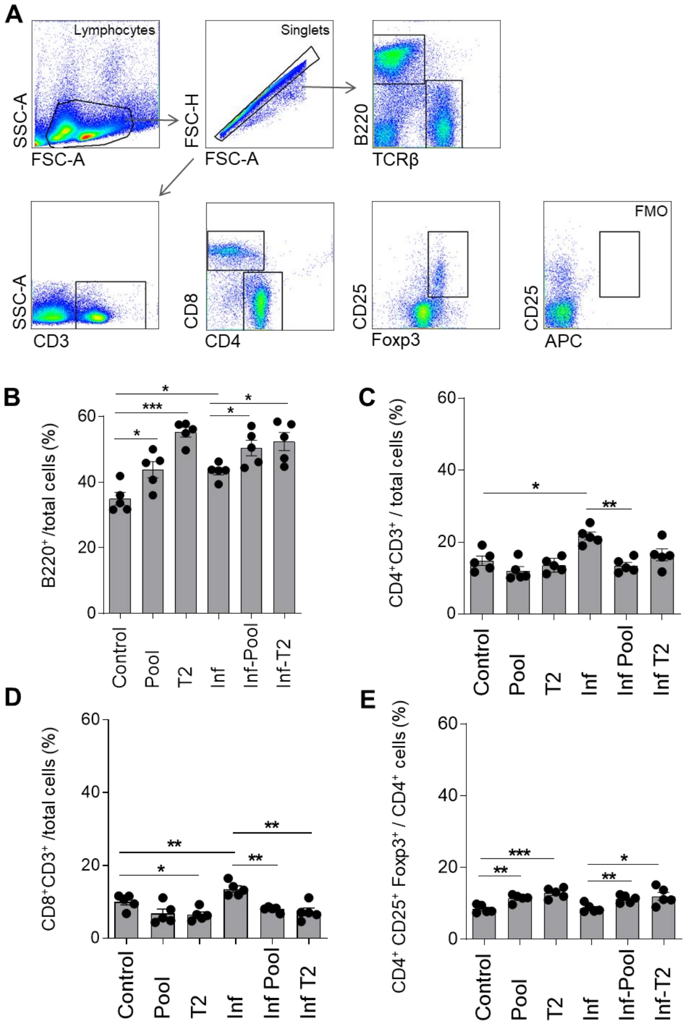

Supplementary Figure 7. Stimulation of the immune system by the Pool and T2 strategies induces differentiation of Treg cells among the CD4 T cell population. Healthy or infected (and treated) mice were immune stimulated, or not, with the Pool or T2 strategy. Splenic lymphocytes subpopulations were analysed at the end of the cognitive behavioural tasks (84 days after the end of CQ treatment), when five mice were randomly chosen per group. Representative gating strategy to identify the populations of B cells ($B220^+$), CD4 T cells ($CD3^+CD4^+$), CD8 T cells ($CD3^+CD8^+$) and Treg cells ($CD3^+CD4^+CD25^+Foxp3^+$) by flow cytometry (**A**). Percentage of B cells (**B**), CD4 T cells (**C**) and CD8 T cells (**D**) per spleen. Percentage of Treg cells among the CD4 T cells population (**E**). Experimental groups: Control (non-infected / non-immune stimulated); Pool (non-infected / Pool-immune stimulated); T2 (non-infected / T2-immune stimulated); Inf (infected / non-immune stimulated); Inf-Pool (infected / Pool-immune stimulated); Inf-T2 (infected / T2-immune stimulated). Data are expressed as mean and s.e.m. **$P < 0.01$; *$P < 0.05$; Unpaired t-test with Mann-Whitney test was used.

Figure S8

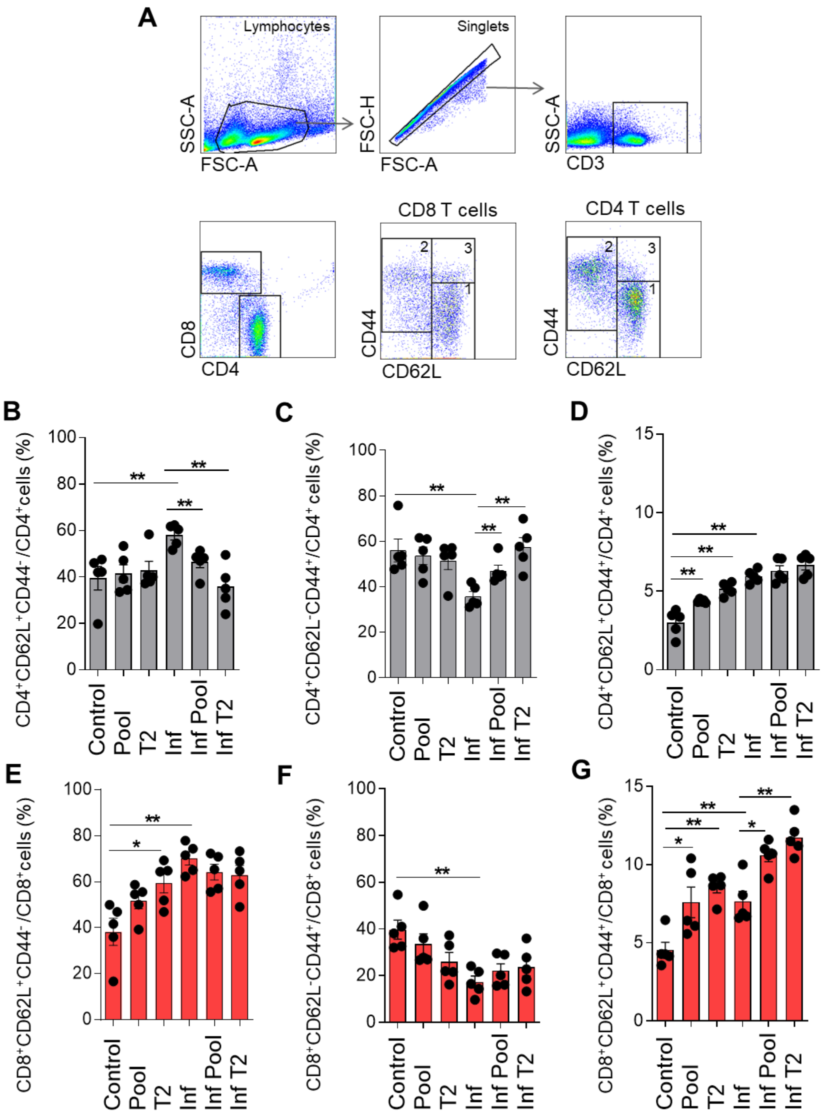

Supplementary Figure 8. Effect of immune stimulation with the Pool and T2 strategies on the activation and memory phenotypes of CD4 and CD8 T cells. Healthy or infected (and treated) mice were immune stimulated, or not, with Pool or T2 strategy. Splenic lymphocyte subpopulations were analysed at the end of the cognitive behavioural tasks (84 days after the end of CQ treatment), when five mice were randomly chosen per group. Representative gating strategy to identify the subpopulations of naïve (gate: 1; $CD44^{-}CD62L^{+}$) (**A**); effector / effector memory (gate: 2; $CD44^{+}CD62L^{-}$) and central memory (gate: 3; $CD44^{+}CD62L^{+}$) CD4 and CD8 T cells by flow cytometry. Percentage of naïve, effector / effector memory and central memory CD4 T cells (**B-D**) and CD8 T cells (**E-G**). Experimental groups: Control (non-infected / non-immune stimulated); Pool (non-infected / Pool-immune stimulated); T2 (non-infected / T2-immune stimulated); Inf (infected / non-immune stimulated); Inf-Pool (infected / Pool-immune stimulated); Inf-T2 (infected / T2-immune stimulated). Data are mean and s.e.m. **$P < 0.01$; *$P < 0.05$; Unpaired t-test with Mann-Whitney test was used.

Table S1

Immune Stimuli	Route	Region	Concentration	Volume	Inoculation
dT vaccine	Subcutaneous	Back	1/5 human dose	100 µl	3
Influenza vaccine	Intramuscular	Quadriceps	1/5 human dose	100 µl	3
pfMSP-3	Subcutaneous	Base Tail	10 µg	100 µl	3
ecLPS	Intraperitoneal	Abdomen	0,1 mg/kg	100 µl	2
Ovalbumin	Subcutaneous Intraperitoneal	Back and Abdomen	50 µg	200 µl	1 s.b.c. 2 i.p.

Supplementary Table 1. Immune stimulus inoculation strategy: route, region, concentration and volume of immunogen and number of injections. s.b.s: subcutaneous and i.p.: intraperitoneal.
